# Analysis of the Use of Sample Size and Effect Size Calculations in a Temporomandibular Disorders Randomised Controlled Trial—Short Narrative Review

**DOI:** 10.3390/jpm14060655

**Published:** 2024-06-19

**Authors:** Grzegorz Zieliński, Piotr Gawda

**Affiliations:** Department of Sports Medicine, Medical University of Lublin, 20-093 Lublin, Poland

**Keywords:** U test, medicine, sample size, Mann–Whitney U test, effect size, TMD, *t* test, statistics, *p*-value, TMDs

## Abstract

Background/Objectives: Temporomandibular disorder (TMD) is the term used to describe a pathology (dysfunction and pain) in the masticatory muscles and temporomandibular joint (TMJ). There is an apparent upward trend in the publication of dental research and a need to continually improve the quality of research. Therefore, this study was conducted to analyse the use of sample size and effect size calculations in a TMD randomised controlled trial. Methods: The period was restricted to the full 5 years, i.e., papers published in 2019, 2020, 2021, 2022, and 2023. The filter article type—“Randomized Controlled Trial” was used. The studies were graded on a two-level scale: 0–1. In the case of 1, sample size (SS) and effect size (ES) were calculated. Results: In the entire study sample, SS was used in 58% of studies, while ES was used in 15% of studies. Conclusions: Quality should improve as research increases. One factor that influences quality is the level of statistics. SS and ES calculations provide a basis for understanding the results obtained by the authors. Access to formulas, online calculators and software facilitates these analyses. High-quality trials provide a solid foundation for medical progress, fostering the development of personalized therapies that provide more precise and effective treatment and increase patients’ chances of recovery. Improving the quality of TMD research, and medical research in general, helps to increase public confidence in medical advances and raises the standard of patient care.

## 1. Introduction

Temporomandibular disorders (TMDs) is the term used to describe pathology (dysfunction and pain) in the masticatory muscles and temporomandibular joint (TMJ). The prevalence of TMDs in the world population is estimated to be 34%. This varies by continent, with South America at 47%, Asia at 33%, Europe at 29% and North America at 26%. Regardless of the continent, women are more likely to suffer from TMDs [1]. The aetiology of TMDs is multifactorial [2]. Factors leading to TMDs are primarily poor TMJ biomechanics and the occurrence of trauma. Perpetuating factors include behavioural, social, emotional, and cognitive factors [3].

The difficulties in treating TMDs arise from this multifactorial aetiology, requiring a comprehensive and individually tailored approach. Combining pharmacological therapy (e.g., analgesics, anti-inflammatory drugs) with physiotherapy, behavioural therapy, and even surgical interventions is often necessary in advanced cases [4,5,6,7]. The effectiveness of treatment is variable and often requires a long-term commitment from both the patient and the treatment team. In the treatment of TMDs, due to the complex aetiology of the condition, a multidisciplinary approach is often utilized. Treatment of TMDs may involve various interventions including psychotherapy, pharmacotherapy, physical therapy, and injection with hyaluronic acid [7,8,9,10]. The estimated cost of treating TMDs in the United States is USD 4 billion [1,11]. 

Randomised controlled trials (RCTs) are considered the gold standard in clinical research and are essential in the treatment of multifactorial conditions, including TMDs [12,13,14]. RCTs minimise error and bias by randomly assigning participants to a treatment or control group. For multifactorial diseases, where multiple factors (genetic, environmental, lifestyle) may influence the development and course of the disease, RCTs allow the assessment of whether a new treatment is effective compared to current treatments or placebo. The results of RCTs are often used to produce clinical guidelines that inform medical practice worldwide. With robust evidence from RCTs, these guidelines can provide the best possible treatment recommendations for multimorbidity [12,13,14,15].

Statistical analysis plays a key role in improving the quality of RCTs by calculating sample size (SS) and effect size (ES) to enable accurate and reliable inferences about the effectiveness of interventions. The SS, calculated at the start of the study, is critical to ensuring that the study has adequate statistical power to detect a significant difference, if any. A sample that is too small may result in the study having insufficient power to detect significant effects, while a sample that is too large may be inefficient in terms of cost and resources [16,17]. Andrade points out that it is unethical to try too large and too little [17]. ES, a measure of the strength of the association between variables, helps to determine whether statistically significant differences are clinically meaningful. For example, Li et al. reviewed the potential benefits of incorporating patient-reported outcomes (PROs) into routine clinical practice for oncology patients [18]. Although they found studies with statistically significant results, the authors note that these had small to moderate ES. This did not allow the authors to confirm that routine incorporation of PROs into clinical practice has a definitive benefit [18]. Another example is Belcher et al., who reviewed the literature on what is known about psychological distress in adults with multiple primary cancers (MPC) [19]. A study reported a potentially significant increase in psychological distress among MPC survivors compared to single cancer survivors, although the ES was small [19]. One of the most popular examples of significance in ES calculations concerns hereditary cardiology. The case is also described by Sullivan and Feinn [20] where a small ES was shown despite significance at *p* < 0.00001 [20,21]. 

There is an apparent upward trend in the publication of dental research and a need to continually improve the quality of research [22]. Therefore, this study was conducted to analyse the use of SS and ES calculations in TMDs RCTs.

## 2. Materials and Methods

It was decided to search the PubMed (National Library of Medicine) [1,23,24] database from 10 January to 30 January 2023 for publications using the acronym: “TMD”. The period was restricted to the full 5 years, i.e., papers published in 2019, 2020, 2021, 2022, and 2023. The time frame of 5 years was chosen due to the designation of this period as the most current scientific literature [25,26,27]. The filter article type—“Randomized Controlled Trial” was used [28]. 

The studies were graded on two-level scale: 0 or 1. In the case of 1, SS and ES were calculated. A score of 0 indicated that no information was found. 

Additionally, each article was assigned a quartile (Q1, Q2, Q3, and Q4) based on the journal’s ranking according to the Scimago Journal & Country Rank [29] for the publication year. In cases where the journal was classified into several different journal ranks, the discipline that best matched the article’s theme was chosen through consensus.

A summary of the PICO standards (population, intervention, comparison, outcome), including inclusion and exclusion criteria, is found in Table 1 [30,31].

## 3. Results

Table 2 presents the qualified papers and the corresponding analysis. In 2019, SS calculations accounted for 46% of the studies, followed by 51%, 56%, 55%, and 81% in 2023. The statistics are less favourable for ES calculations, with 4% in 2019, and subsequently 21%, 19%, 15%, and 10% in 2023. Across the entire sample, SS was used in 58% of the studies, while ES was used in 15%. The simultaneous use of SS and ES occurred in 4% of the studies in 2019, 18% in 2020, and then 17%, 12%, and 10% in subsequent years. In the entire surveyed group, this amounted to 13%

**Figure 1:**
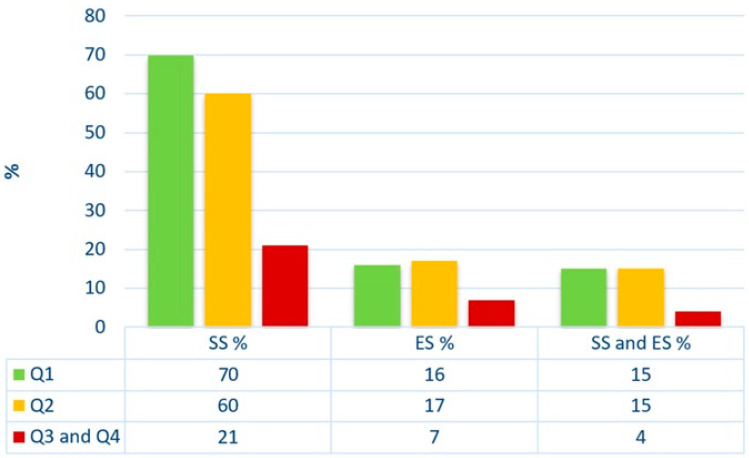
Analysis of the usage of SS, ES, and the combined use of SS and ES depending on the quartile determined by Scimago Journal & Country Rank. SS—sample size calculations; ES—effect size; Q—Quartiles according to Scimago Journal & Country Rank.

An analysis of the usage of SS, ES, and the combined use of SS and ES in scholarly papers was conducted based on the quartile assigned to the journal according to the Scimago Journal & Country Rank. The sample analysed consisted of 82 papers published in Q1, 53 papers published in Q2, 28 papers in Q3, and 1 in Q4. It was decided to combine quarters Q3 and Q4. A similar percentage of papers using SS, ES, and the combined use of SS and ES was shown to be published in journals placed in Q1 and Q2. However, the lowest usage of SS, ES, and the combined use of SS and ES was indicated in papers published in quarters Q3 and Q4. The results are presented in Table 2 and Figure 1. 

## 4. Discussion

There is an evident upward trend in the publication of dental research, highlighting the ongoing need to enhance research quality [22]. Consequently, this study was conducted to analyse the use of SS and ES calculations in TMDs RCTs. The findings indicate a continued necessity for incorporating SS and ES calculations in research methodologies. However, few authors currently perform these calculations.

The added value of including information on SS and ES calculation in standardized places is a key element of reporting research results. The first important reason is to ensure the transparency and replicability of the research. Detailed information on SS allows readers to accurately assess the statistical power of the analysis so that the study can be repeated to confirm the results. In addition, the inclusion of ES data helps to understand the practical significance of the results obtained, which is important for the scientific community and practitioners.

In standardized sections, such as Methodology and Statistics, this information is readily accessible and comprehensible to readers. Additionally, the clear presentation of this data helps prevent the misinterpretation of results that can arise from insufficient information about the study conditions. Standardized reporting of this information also facilitates research for other scholars, particularly those conducting literature reviews and meta-analyses. In the subsequent part of this work, we demonstrate the methods for calculating SS and ES. 

Numerous formulas are available for SS calculation, some of which are presented in Table 3. Other formulas can be found in the articles of Charan and Biswas [16], Noordzij et al. [195] and Das et al. [196].

Parameters such as *p*-value, power, etc., must be included in calculations and formulae. It is therefore worth noting that the most commonly used values are 0.05 (5%) and 0.01 (1%) [201,202]. Statistical power refers to the likelihood of identifying a significant effect or difference, if present, within the population [203,204]. The power level of a test is usually set at 0.80 [205,206,207]. 

It is necessary to explain why sample size calculations should be conducted for each study. For example, in the studies on TMDs RCTs, the following sample sizes were used: Calixtre et al., n = 61 [42]; Huth et al., n = 40 [66]; Şahin et al., n = 50 [74] Serrano-Hernanz et al., n = 72 [86]; Gikić et al., n = 84 [114]. This example demonstrates that although the field pertains to dental patients, the number of participants appropriate for each study will depend on the study design.

The SS determination also applies to retrospective studies, but the process may be somewhat more complex. When planning SS for retrospective studies, it is important to understand that limited data availability may affect the ability to estimate SS. To estimate SS in retrospective studies, the formulas in Table 3 can be used, but Johnston et al. have also provided an SS computation in descriptive retrospective burden investigations [208].

A different approach is presented by Kim and Seo in the form of post hoc power analyses in retrospective studies [209]. In retrospective studies, it is often not possible to change the SS because the data have already been collected. Post hoc power analysis can help to understand how robust the observed effects are in the context of already collected data when SS calculation is difficult.

Table 4 and Table 5 show the main formulas for ES. Other formulas can be found in the work of Lakens [210], Tomczak and Tomczak [211], and Fritz [212]. 

Let us consider examples from studies analysed in this research. In the study by Calixtre et al., an effect size of d > 0.8 was observed, which further validated the obtained results [42]. In the study by Huth et al., the ES demonstrated that aqualizer system-based occlusal splints led to a better improvement in TMJ pain with maximum opening compared to chin point guidance-based occlusal splints (d = 0.9; chin point guidance d = 0.13) [66]. Here, the ES helped differentiate the outcomes. In the study by Şahin et al., which aimed to compare the effects of 4 weeks of exercise combined with ischemic compression and exercise alone in patients with TMDs [74], the ES was small for maximum assisted mouth opening (ES = 0.27) and moderate for painless mouth opening (ES = 0.51) [74]. This indicates that despite the significance, the phenomenon requires further research.

An extension of the concept of ES Cohen’s d was proposed by Sawilowsky in 2009. He added the ES categories “Very Small, Very Large, and Huge” [237]. This extension aims to increase the statistical power and reduce the risk of erroneous data interpretation (Table 6).

In the main text of the paper, in addition to tables with formulas for SS and ES, there are tables with free calculators for these values available online (Table 6) as a form of supplement to the paper Serdar et al. [238] or Charan and Biswas [16]. However, there were no academic calculators or government websites to help with the analyses in the above studies, so Table 7 was created. In addition to the online tools, statistical software tools such as G*Power, R, GraphPad Prism, and SPSS are also available to assist in the analysis of the above variables. Based on the relevance of SS and ES, and the accessibility of their calculation (formulas, online calculators, software tools), it is recommended that they be included in medical research.

Clinical studies on TMDs require careful planning and analysis to provide meaningful insights for clinical practice. Key aspects of these studies include calculations regarding SS and ES, which help ensure the reliability and utility of the results. The ES is a crucial indicator for assessing the clinical significance of the findings and can be expressed in various ways depending on the study’s characteristics and outcomes. Conclusions drawn from these analyses can aid in delivering better treatment strategies for patients with TMDs, by enhancing the effectiveness and precision of therapeutic interventions and improving understanding of their impact on patients’ quality of life.

Given the numerous tools available to researchers for performing SS and ES calculations, it is essential to explore why so few studies address this topic. Our discussion has highlighted several studies emphasizing the importance of SS and ES calculations [20,197,209,211]. The lack of these calculations may be attributed to a multifaceted issue.

One factor could be the lack of adequate training in statistics, leading to an incomplete understanding of SS and ES calculation methods. The significance of collaboration between medical specialists and statisticians was observed by Sprent in 2003 [239]. Another issue relates to sample size, which can be impacted by the application of new therapies [57,190] and participants’ concerns about potential adverse effects, resulting in lower recruitment numbers [240,241]. Challenges in collaboration, such as conducting single-centre studies with selected and hard-to-recruit patients due to various inclusion and exclusion criteria, further complicate this issue [58,191].

The examples mentioned above suggest that the infrequent use of SS and ES calculations may be linked to publication pressure. Kearney et al., who investigated this phenomenon in medical research, summarized their findings as follows: “Pressure in the world of academic medicine to publish contributes to the potential for research misconduct and authorship misrepresentation” [242]. This summary also aptly explains our results.

## 5. Conclusions

Quality should improve as research increases. One factor that influences quality is the level of statistics. SS and ES calculations provide a basis for understanding the results obtained by the authors. SS post hoc calculations should also be used in retrospective studies. Access to formulas, online calculators and software facilitates these analyses. High-quality trials provide a solid foundation for medical progress, fostering the development of personalized therapies that provide more precise and effective treatment and increase patients’ chances of recovery. Improving the quality of TMD research, and medical research in general, helps to increase public confidence in medical advances and raises the standard of patient care.

## Figures and Tables

**Table 1:** PICO summary of inclusion and exclusion criteria.

	Inclusion	Exclusion
Patient		
	Adult and Pediatric population	
Intervention		
	Treatment and investigation of TMDs	
Outcome		
	Classification of the research as a randomised controlled trial in the PubMed database.	
Comparison		
	TMDs vs. Health Subject TMDs vs. TMDs	
Study Design		
		Clinical Trial Narrative Review Systematic Articles Meta-analysis Opinions Case reports or series patients Animal or biomechanical studies Publications in a language other than English Post-conference abstracts

TMDs—Temporomandibular disorders.

**Table 2:** Presentation of results on the use of sample size calculations and effect size in the analysed studies.

		2019				2020				2021				2022				2023		
No.	ID	Q	SS	ES	ID	Q	SS	ES	ID	Q	SS	ES	ID	Q	SS	ES	ID	Q	SS	ES
1	[32]	Q1	1	0	[33]	Q3	0	0	[34]	Q2	0	0	[35]	Q3	0	0	[36]	Q2	1	0
2	[37]	Q2	1	0	[38]	Q3	0	0	[39]	Q3	0	0	[40]	Q4	0	0	[41]	Q1	1	0
3	[42]	Q1	1	1	[43]	Q2	1	1	[44]	Q1	0	0	[45]	Q2	1	1	[46]	Q1	1	0
4	[47]	Q1	0	0	[48]	Q1	1	0	[49]	Q1	1	0	[50]	Q1	0	0	[51]	Q1	1	0
5	[52]	Q3	0	0	[53]	Q1	0	0	[54]	Q2	1	0	[55]	Q3	1	0	[56]	Q1	1	0
6	[57]	Q1	0	0	[58]	Q2	0	0	[59]	Q2	0	0	[60]	Q1	1	0	[61]	Q1	1	0
7	[62]	Q1	1	0	[63]	Q1	1	0	[64]	Q2	0	0	[65]	Q2	0	0	[66]	Q1	1	1
8	[67]	Q1	1	0	[68]	Q3	0	0	[69]	Q3	0	0	[70]	Q3	0	1	[71]	Q1	1	0
9	[72]	Q1	0	0	[73]	Q1	0	1	[74]	Q1	1	1	[75]	Q2	0	0	[76]	Q1	0	0
10	[77]	Q1	1	0	[78]	Q1	1	0	[79]	Q3	1	0	[80]	Q2	1	0	[81]	Q1	0	0
11	[82]	Q1	0	0	[83]	Q2	0	0	[84]	Q3	1	1	[85]	Q3	0	0	[86]	Q1	1	1
12	[87]	Q1	0	0	[88]	Q1	0	0	[89]	Q3	0	0	[90]	Q1	0	0	[91]	Q1	1	0
13	[92]	Q1	1	0	[93]	Q2	1	1	[94]	Q3	1	0	[95]	Q3	1	0	[96]	Q1	1	0
14	[97]	Q2	1	0	[98]	Q1	0	0	[99]	Q1	0	0	[100]	Q1	1	0	[101]	Q1	1	0
15	[102]	Q2	0	0	[103]	Q1	1	1	[104]	Q2	0	1	[105]	Q1	1	0	[106]	Q1	0	0
16	[107]	Q2	1	0	[108]	Q2	0	0	[109]	Q3	0	0	[110]	Q2	1	0	[111]	Q1	1	1
17	[112]	Q2	0	0	[113]	Q1	0	0	[114]	Q1	1	1	[115]	Q2	1	1	[116]	Q1	1	0
18	[117]	Q3	0	0	[118]	Q2	0	0	[119]	Q2	1	0	[120]	Q2	1	0	[121]	Q1	1	0
19	[122]	Q1	1	0	[123]	Q3	0	0	[124]	Q1	1	0	[125]	Q2	1	0	[126]	Q1	1	0
20	[127]	Q3	0	0	[128]	Q1	1	0	[129]	Q2	0	0	[130]	Q2	1	0	[131]	Q1	0	0
21	[132]	Q1	0	0	[133]	Q1	1	0	[134]	Q2	0	0	[135]	Q1	1	1	[136]	Q1	1	0
22	[137]	Q2	0	0	[138]	Q2	1	0	[139]	Q1	1	0	[140]	Q3	0	0	[141]	Q1	1	0
23	[142]	Q1	1	0	[143]	Q2	1	1	[144]	Q1	0	0	[145]	Q2	1	0	[146]	Q1	0	0
24	[147]	Q2	0	0	[148]	Q3	0	0	[149]	Q2	1	0	[150]	Q3	0	0	[151]	Q1	1	0
25					[152]	Q2	1	0	[153]	Q3	0	0	[154]	Q2	1	1	[155]	Q2	1	0
26					[156]	Q1	1	1	[157]	Q2	1	0	[158]	Q3	0	0	[159]	Q1	1	0
27					[160]	Q2	0	0	[161]	Q1	1	0	[162]	Q2	0	0	[163]	Q1	1	0
28					[164]	Q1	1	1	[165]	Q2	1	1	[166]	Q1	0	0	[167]	Q1	1	0
29					[168]	Q1	0	0	[169]	Q2	1	0	[170]	Q2	0	0	[171]	Q1	1	0
30					[172]	Q1	1	0	[173]	Q2	1	1	[174]	Q2	1	0	[175]	Q1	1	0
31					[176]	Q2	1	0	[177]	Q1	1	0	[178]	Q2	1	0	[179]	Q1	1	0
32					[180]	Q1	1	0	[181]	Q3	0	0	[182]	Q3	1	0				
33					[183]	Q3	0	0	[184]	Q1	1	0	[185]	Q2	0	0				
34					[186]	Q1	1	1	[187]	Q1	1	0								
35					[188]	Q2	1	0	[189]	Q1	1	1								
36					[190]	Q2	0	0	[191]	Q3	0	0								
37					[192]	Q1	0	0												
38					[193]	Q2	1	0												
39					[194]	Q2	1	0												
Total In Year			11	1			20	8			20	7			18	5			26	3
%			46	4			51	21			56	19			55	15			84	10
																				
		Total SS calculation									n	95			58	%				
		Total ES calculation									n	24			15	%				

SS—sample size calculations; ES—effect size; Q—Quartiles according to Scimago Journal & Country Rank.

**Table 3:** Example formulas that can be used to calculate the SS.

	Name	Formula for Sample Size	Example of Use
1	*Formula for Proportions (Binomial)*	$n=\frac{Z^{2}p(1-p)}{E^{2}}$	The formula for proportions (binomial), can be used to study the effectiveness of treatments or medical interventions, to study the incidence or prevalence of diseases and to study the efficacy of a drug, etc.
2	*Formula for Mean (Homogeneity)*	$n=\frac{Z^{2}\sigma^{2}}{E^{2}}$	The mean (homogeneity) formula can be used in studies that measure health parameters (hormone levels, blood pressure, blood sugar levels, etc.), the effectiveness of diagnostic or therapeutic interventions, and studies that focus on average treatment outcomes.

n—sample size; Z—confidence level; p—estimated population proportion; E—margin of error; $\sigma^{2}$ —value is the population variance; Based on publications [16,197,198,199,200].

**Table 4:** Example formulas that can be used to calculate the ES.

	Formula For Effect Size	Example of Use
1	$d=\frac{t}{\sqrt{n}}$	ES for Student’s *t*-test
2	$d=\frac{{\bar{X}}_{1}-{\bar{X}}_{2}}{s}$	ES for Student’s *t*-test
3	$g=d(1-\frac{3}{4n-9})$	Hedges’s g correction for bias (Student’s *t*-test) recommended when *n* < 50
4	$\delta =\frac{{\bar{X}}_{1}-{\bar{X}}_{2}}{s_{2}}$	Glass’s δ
5	$r=\frac{Z}{\sqrt{n}}$	ES for the Mann–Whitney U test or Wilcoxon test
6	$r=\frac{2({\bar{R}}_{1}-{\bar{R}}_{2})}{n_{1}{+n}_{2}}$	ES for the Mann–Whitney U test or Wilcoxon test
7	$d=\frac{2r}{\sqrt{1-r^{2}}}$	Formula for converting r into Cohen’s d effect size
8	$\varphi =\sqrt{\frac{x^{2}}{n}}$	ES for the Chi-Squared test
9	$V=\sqrt{\frac{x^{2}}{n_{min}(R-1)(C-1)}}$	ES for the Chi-Squared test
10	$\eta 2=\frac{H-k+1}{n-1}$	ES for a Kruskal–Wallis test

Table based on publications [210,211,212,213,214,215,216,217,218,219]. d—Cohen’s index; t—value of Student’s *t*-test; n—sample size; ${\bar{X}}_{1}$ and ${\bar{X}}_{2}$ —the average values for the two groups; s— the pooled standard deviation; $s_{2}$ —the standard deviation of the second group; φ—Phi effect size; δ —Glass’s index; r—correlation coefficient (−1.00 to 1.00); z—value of U-test; ${\bar{R}}_{1}$ and ${\bar{R}}_{2}$ —mean range for group 1 and group 2; $n_{1}$ and $n_{2}$ —represent the number of observations in each group; U—stands for the Mann–Whitney; V—Cramer’s V effect size; x^2^—the chi-squared statistic; R—number of rows; C—number of columns; n_min_—the minimum number of observations (the minimum value among two values: the number of rows and the number of columns in a given contingency table); η2 —index; H—value of H-test; k—number of groups. chi-squared test statistic.

**Table 5:** The most commonly used coefficients for effect size, based on publications.

Coefficients	Small	Medium	Large	What is/When to Use	
Cliff’s δ	0.15	0.33	0.47	It is a measure that compares two groups in the case of ordinal or ranked variables. It is used to assess the difference in distribution between two groups, but unlike many other effect measures, it is more robust to sample imbalance, data skewness and non-linear relationships.	
Cohen’s d	0.20	0.50	0.80	This measure works best for comparisons between two groups, for example, an experimental group and a control group (comparison of two groups or differences between averages).	
Cohen’s d	0.15	0.40	0.75	Brydges’ recommendation in gerontology [220].	
Cohen’s d	0.25	0.55	0.95	Gaeta and Brydges’ recommendation in audiology and speech-language pathology [221].	
Cohen’s d	0.15	0.36	0.65	Lovakov and Agadullina’s recommendation in social psychology and sub-disciplines within social psychology [222].	
Cohen’s g	0.05	0.15	0.25	Cohen’s g is a less common variant of Cohen’s d and is used to measure the difference between 2 groups (for example in McNemar’s test).	
Cohen’s f	0.10	0.25	0.40	It is used when there is a comparative analysis of more than two groups.	
Cohen’s ω	0.10	0.30	0.50	Cohen’s ω is used in regression analyses, particularly for linear regression, to measure how strongly factors are related.	
Cramér’s V and phi (φ)				df	Cramér’s V—it is used to assess the strength of the relationship between 2 or more categorical factors in tables of different sizes, allowing comparison across different contingency table sizes. It s a more general measure applicable to tables of different dimensions (different numbers of rows and columns). phi (φ)—specifically used for 2 × 2 contingency tables, ϕ measures the strength of the connection between categorical variables in contingency tables. It focuses on tables of a fixed size, making it suitable for more specific contexts like two-factor analysis in medical studies or qualitative research.
	0.1	0.3	0.5	1	
	0.07	0.21	0.35	2	
	0.06	0.17	0.29	3	
	0.05	0.15	0.25	4	
	0.04	0.13	0.22	5	
Glass’s δ	0.20	0.50	0.80	It is used in the context of experimental analysis, where one group is treated as the control group and the other as the experimental group.	
Hedges’ g	0.20	0.50	0.80	This coefficient is a measure of ES similar to Cohen’s d, but with a correction for SS. Used for intergroup analyses with small samples.	
Hedges’ g	0.15	0.40	0.75	Brydges recommendation in gerontology [220].	
Hedges’ g	0.25	0.55	0.95	Gaeta and Brydges’ recommendation in audiology and speech-language pathology [221].	
Pearson’s r	0.10	0.30	0.50	It is used to measure the strength and direction of a relationship between 2 continuous factors. It is employed to quantify the intensity and orientation of a connection between 2 continuous variables.	
Pearson’s r	0.10	0.20	0.30	Brydges’ recommendation in gerontology [220].	
Pearson’s r	0.25	0.40	0.65	Gaeta and Brydges’ recommendation in audiology and speech-language pathology [221].	
Pearson’s r	0.12	0.24	0.41	Lovakov and Agadullina’s recommendation in social psychology and related disciplines [222].	
Pearson’s r	0.10	0.20	0.30	Gignac and Szodorai’s recommendation [223].	
Odds Ratio	1.44	2.48	4.27	Odds ratio is a measure used in statistics, especially in epidemiology and other areas of medical research, to determine the strength of the relationship between 2 variables, usually in the context of a case–control study.	
Odds Ratio	1.68	3.47	6.71	Recommended by Chen et al. [224].	
η^2^	0.01	0.06	0.14	It is a measure of ES used mainly in the analysis of variance (ANOVA). It is used to assess the strength of the connection between the independent and dependent variables when we have more than two groups of data.	
ω^2^	0.01	0.06	0.14	It is a measure of ES in the analysis of variance (ANOVA). This is a more sophisticated measure that takes into account the number of groups and the number of observations in each group. It is considered to be a more accurate and less biased measure of effect size in ANOVA than η^2^.	

Table based on [210,213,220,221,222,223,224,225,226,227,228,229,230,231,232,233,234,235,236].

**Table 6:** Extension of Cohen’s d effect size concept proposed by Sawilowsky.

	Very Small	Small	Medium	Large	Very Large	Huge
Cohen’s d		0.20	0.50	0.80		
Sawilowsky	0.01				1.20	2.00

Table based on [227,237].

**Table 7:** Online sample size and effect size calculators.

Institution	Link Accessed on 16 May 2024
Australian Bureau of Statistics	https://www.abs.gov.au/websitedbs/d3310114.nsf/home/sample+size+calculator
Cambridge University	https://www.cem.org/effect-size-calculator
Harvard University	http://hedwig.mgh.harvard.edu/sample_size/size.html
Johns Hopkins University	http://www.rad.jhmi.edu/jeng/javarad/samplesize/
Missouri State University	https://www.missouristate.edu/RStats/mote-effect-size-calculator.htm
Universität Wien	https://homepage.univie.ac.at/robin.ristl/samplesize.php
Universiti Sains Malaysia	https://medic.usm.my/biostat/en/articles/118-sample-size-calculator
University College London	https://www.ucl.ac.uk/child-health/short-courses-events/about-statistical-courses/sample-size-estimation-and-power-calculations
University of British Columbia	https://www.stat.ubc.ca/~rollin/stats/ssize/
University of California, San Francisco	https://sample-size.net/means-sample-sizeclustered/
University of Colorado	https://lbecker.uccs.edu/
University of Michigan	https://csg.sph.umich.edu/abecasis/cats/gas_power_calculator/

## References

[1] Zieliński G., Pająk-Zielińska B., Ginszt M. (2024). A Meta-Analysis of the Global Prevalence of Temporomandibular Disorders. J. Clin. Med..

[2] Chisnoiu A.M., Picos A.M., Popa S., Chisnoiu P.D., Lascu L., Picos A., Chisnoiu R. (2015). Factors Involved in the Etiology of Temporomandibular Disorders—A Literature Review. Clujul Med..

[3] Sharma S., Gupta D.S., Pal U.S., Jurel S.K. (2011). Etiological Factors of Temporomandibular Joint Disorders. Natl. J. Maxillofac. Surg..

[4] Wroclawski C., Mediratta J.K., Fillmore W.J. (2023). Recent Advances in Temporomandibular Joint Surgery. Medicina.

[5] Dimitroulis G. (2018). Management of Temporomandibular Joint Disorders: A Surgeon’s Perspective. Aust. Dent. J..

[6] Ouanounou A., Goldberg M., Haas D.A. (2017). Pharmacotherapy in Temporomandibular Disorders: A Review. J. Can. Dent. Assoc..

[7] Penlington C., Otemade A.A., Bowes C., Taylor G., Waterhouse P., Ohrbach R. (2019). Psychological Therapies for Temporomandibular Disorders (TMD). Cochrane Database Syst. Rev..

[8] Abouelhuda A.M., Khalifa A.K., Kim Y.-K., Hegazy S.A. (2018). Non-Invasive Different Modalities of Treatment for Temporomandibular Disorders: Review of Literature. J. Korean Assoc. Oral Maxillofac. Surg..

[9] Ginszt M., Zieliński G., Berger M., Szkutnik J., Bakalczuk M., Majcher P. (2020). Acute Effect of the Compression Technique on the Electromyographic Activity of the Masticatory Muscles and Mouth Opening in Subjects with Active Myofascial Trigger Points. Appl. Sci..

[10] Agostini F., Ferrillo M., Bernetti A., Finamore N., Mangone M., Giudice A., Paoloni M., de Sire A. (2023). Hyaluronic Acid Injections for Pain Relief and Functional Improvement in Patients with Temporomandibular Disorders: An Umbrella Review of Systematic Reviews. J. Oral Rehabil..

[11] Matheson E.M., Fermo J.D., Blackwelder R.S. (2023). Temporomandibular Disorders: Rapid Evidence Review. Am. Fam. Physician.

[12] Hariton E., Locascio J.J. (2018). Randomised Controlled Trials—The Gold Standard for Effectiveness Research. BJOG Int. J. Obstet. Gynaecol..

[13] Kabisch M., Ruckes C., Seibert-Grafe M., Blettner M. (2011). Randomized Controlled Trials. Dtsch. Ärztebl. Int..

[14] Di Francesco F., Lanza A., Di Blasio M., Vaienti B., Cafferata E.A., Cervino G., Cicciù M., Minervini G. (2022). Application of Botulinum Toxin in Temporomandibular Disorders: A Systematic Review of Randomized Controlled Trials (RCTs). Appl. Sci..

[15] Weiss C.O., Varadhan R., Puhan M.A., Vickers A., Bandeen-Roche K., Boyd C.M., Kent D.M. (2014). Multimorbidity and Evidence Generation. J. Gen. Intern. Med..

[16] Charan J., Biswas T. (2013). How to Calculate Sample Size for Different Study Designs in Medical Research?. Indian J. Psychol. Med..

[17] Andrade C. (2020). Sample Size and Its Importance in Research. Indian J. Psychol. Med..

[18] Li D., Huang Q., Zhang W., Yuan C., Wu F. (2023). Effects of Routine Collection of Patient-Reported Outcomes on Patient Health Outcomes in Oncology Settings: A Systematic Review. Asia-Pac. J. Oncol. Nurs..

[19] Belcher S.M., Hausmann E.A., Cohen S.M., Donovan H.S., Schlenk E.A. (2017). Examining the Relationship between Multiple Primary Cancers and Psychological Distress: A Review of Current Literature. Psychooncology.

[20] Sullivan G.M., Feinn R. (2012). Using Effect Size—Or Why the P Value Is Not Enough. J. Grad. Med. Educ..

[21] Bartolucci A.A., Tendera M., Howard G. (2011). Meta-Analysis of Multiple Primary Prevention Trials of Cardiovascular Events Using Aspirin. Am. J. Cardiol..

[22] Zieliński G., Gawda P. (2024). Surface Electromyography in Dentistry—Past, Present and Future. J. Clin. Med..

[23] Booth A. (2016). Over 85% of Included Studies in Systematic Reviews Are on MEDLINE. J. Clin. Epidemiol..

[24] Halladay C.W., Trikalinos T.A., Schmid I.T., Schmid C.H., Dahabreh I.J. (2015). Using Data Sources beyond PubMed Has a Modest Impact on the Results of Systematic Reviews of Therapeutic Interventions. J. Clin. Epidemiol..

[25] Poojary S.A., Bagadia J.D. (2014). Reviewing Literature for Research: Doing It the Right Way. Indian J. Sex. Transm. Dis. Aids.

[26] Kruse C.S., Beane A. (2018). Health Information Technology Continues to Show Positive Effect on Medical Outcomes: Systematic Review. J. Med. Internet Res..

[27] Luft J.A., Jeong S., Idsardi R., Gardner G. (2022). Literature Reviews, Theoretical Frameworks, and Conceptual Frameworks: An Introduction for New Biology Education Researchers. CBE Life Sci. Educ..

[28] https://pubmed.ncbi.nlm.nih.gov/?Term=TMD&filter=pubt.Randomizedcontrolledtrial&filter=years.2019-2023.

[29] Scimago Journal & Country Rank. https://www.scimagojr.com/.

[30] Brown D. (2020). A Review of the PubMed PICO Tool: Using Evidence-Based Practice in Health Education. Health Promot. Pract..

[31] Stone P.W. (2002). Popping the (PICO) Question in Research and Evidence-Based Practice. Appl. Nurs. Res..

[32] Nagata K., Hori S., Mizuhashi R., Yokoe T., Atsumi Y., Nagai W., Goto M. (2019). Efficacy of Mandibular Manipulation Technique for Temporomandibular Disorders Patients with Mouth Opening Limitation: A Randomized Controlled Trial for Comparison with Improved Multimodal Therapy. J. Prosthodont. Res..

[33] Chellappa D., Thirupathy M. (2020). Comparative Efficacy of Low-Level Laser and TENS in the Symptomatic Relief of Temporomandibular Joint Disorders: A Randomized Clinical Trial. Indian J. Dent. Res. Off. Publ. Indian Soc. Dent. Res..

[34] Urbański P., Trybulec B., Pihut M. (2021). The Application of Manual Techniques in Masticatory Muscles Relaxation as Adjunctive Therapy in the Treatment of Temporomandibular Joint Disorders. Int. J. Environ. Res. Public Health.

[35] Dib-Zakkour J., Flores-Fraile J., Montero-Martin J., Dib-Zakkour S., Dib-Zaitun I. (2022). Evaluation of the Effectiveness of Dry Needling in the Treatment of Myogenous Temporomandibular Joint Disorders. Medicina.

[36] Santana-Penín U., Santana-Mora U., López-Solache A., Mora M.J., Collier T., Pocock S.J., Lorenzo-Franco F., Varela-Centelles P., López-Cedrún J.L. (2023). Remodeling Dental Anatomy vs Sham Therapy for Chronic Temporomandibular Disorders. A Placebo-Controlled Randomized Clinical Trial. Ann. Anat. Anat. Anz. Off. Organ Anat. Ges..

[37] Oliveira S.S.I., Pannuti C.M., Paranhos K.S., Tanganeli J.P.C., Laganá D.C., Sesma N., Duarte M., Frigerio M.L.M.A., Cho S. (2019). Effect of Occlusal Splint and Therapeutic Exercises on Postural Balance of Patients with Signs and Symptoms of Temporomandibular Disorder. Clin. Exp. Dent. Res..

[38] Macedo De Sousa B., López-Valverde N., López-Valverde A., Caramelo F., Flores Fraile J., Herrero Payo J., Rodrigues M.J. (2020). Different Treatments in Patients with Temporomandibular Joint Disorders: A Comparative Randomized Study. Medicina.

[39] Kaya D.I., Ataoglu H. (2021). Botulinum Toxin Treatment of Temporomandibular Joint Pain in Patients with Bruxism: A Prospective and Randomized Clinical Study. Niger. J. Clin. Pract..

[40] Pihut M., Zarzecka-Francica E., Gala A. (2022). Physiotherapeutic Rehabilitation of Adolescent Patients with Temporomandibular Disorders. Folia Med. Cracov..

[41] Al-Quisi A.F., Jamil F.A., Abdulhadi B.N., Muhsen S.J. (2023). The Reliability of Using Light Therapy Compared with LASER in Pain Reduction of Temporomandibular Disorders: A Randomized Controlled Trial. BMC Oral Health.

[42] Calixtre L.B., Oliveira A.B., de Sena Rosa L.R., Armijo-Olivo S., Visscher C.M., Alburquerque-Sendín F. (2019). Effectiveness of Mobilisation of the Upper Cervical Region and Craniocervical Flexor Training on Orofacial Pain, Mandibular Function and Headache in Women with TMD. A Randomised, Controlled Trial. J. Oral Rehabil..

[43] Delgado de la Serna P., Plaza-Manzano G., Cleland J., Fernández-de-las-Peñas C., Martín-Casas P., Díaz-Arribas M.J. (2020). Effects of Cervico-Mandibular Manual Therapy in Patients with Temporomandibular Pain Disorders and Associated Somatic Tinnitus: A Randomized Clinical Trial. Pain Med..

[44] Aisaiti A., Zhou Y., Wen Y., Zhou W., Wang C., Zhao J., Yu L., Zhang J., Wang K., Svensson P. (2021). Effect of Photobiomodulation Therapy on Painful Temporomandibular Disorders. Sci. Rep..

[45] Tobe S., Ishiyama H., Nishiyama A., Miyazono K., Kimura H., Fueki K. (2022). Effects of Jaw-Opening Exercises with/without Pain for Temporomandibular Disorders: A Pilot Randomized Controlled Trial. Int. J. Environ. Res. Public Health.

[46] Benli M., Olson J., Huck O., Özcan M. (2023). A Novel Treatment Modality for Myogenous Temporomandibular Disorders Using Aromatherapy Massage with Lavender Oil: A Randomized Controlled Clinical Trial. CRANIO^®^.

[47] Huttunen J., Qvintus V., Suominen A.L., Sipilä K. (2019). Role of Psychosocial Factors on Treatment Outcome of Temporomandibular Disorders. Acta Odontol. Scand..

[48] Wänman A., Marklund S. (2020). Treatment Outcome of Supervised Exercise, Home Exercise and Bite Splint Therapy, Respectively, in Patients with Symptomatic Disc Displacement with Reduction: A Randomised Clinical Trial. J. Oral Rehabil..

[49] De La Torre Canales G., Câmara-Souza M.B., Poluha R.L., Grillo C.M., Conti P.C.R., Sousa M.D.L.R.D., Rodrigues Garcia R.C.M., Rizzatti-Barbosa C.M. (2021). Botulinum Toxin Type A and Acupuncture for Masticatory Myofascial Pain: A Randomized Clinical Trial. J. Appl. Oral Sci. Rev. FOB.

[50] van der Wal A., Michiels S., Van de Heyning P., Gilles A., Jacquemin L., Van Rompaey V., Braem M., Visscher C.M., Topsakal V., Truijen S. (2022). Reduction of Somatic Tinnitus Severity Is Mediated by Improvement of Temporomandibular Disorders. Otol. Neurotol..

[51] Aguiar A.D.S., Moseley G.L., Bataglion C., Azevedo B., Chaves T.C. (2023). Education-Enhanced Conventional Care versus Conventional Care Alone for Temporomandibular Disorders: A Randomized Controlled Trial. J. Pain.

[52] Ramakrishnan S.N., Aswath N. (2019). Comparative Efficacy of Analgesic Gel Phonophoresis and Ultrasound in the Treatment of Temporomandibular Joint Disorders. Indian J. Dent. Res. Off. Publ. Indian Soc. Dent. Res..

[53] Bergmann A., Edelhoff D., Schubert O., Erdelt K.-J., Pho Duc J.-M. (2020). Effect of Treatment with a Full-Occlusion Biofeedback Splint on Sleep Bruxism and TMD Pain: A Randomized Controlled Clinical Trial. Clin. Oral Investig..

[54] Santana-Mora U., López-Cedrún J., Suárez-Quintanilla J., Varela-Centelles P., Mora M.J., Da Silva J.L., Figueiredo-Costa F., Santana-Penín U. (2021). Asymmetry of Dental or Joint Anatomy or Impaired Chewing Function Contribute to Chronic Temporomandibular Joint Disorders. Ann. Anat.—Anat. Anz..

[55] Poorna T.A., John B., EK J., Rao A. (2022). Comparison of the Effectiveness of Soft and Hard Splints in the Symptomatic Management of Temporomandibular Joint Disorders: A Randomized Control Study. Int. J. Rheum. Dis..

[56] Tanhan A., Ozer A.Y., Polat M.G. (2023). Efficacy of Different Combinations of Physiotherapy Techniques Compared to Exercise and Patient Education in Temporomandibular Disorders: A Randomized Controlled Study. CRANIO^®^.

[57] De Riu G., Vaira L.A., Carta E., Meloni S.M., Sembronio S., Robiony M. (2019). Bone Marrow Nucleated Cell Concentrate Autograft in Temporomandibular Joint Degenerative Disorders: 1-Year Results of a Randomized Clinical Trial. J. Cranio-Maxillofac. Surg..

[58] Li W., Wu J. (2020). Treatment of Temporomandibular Joint Disorders by Ultrashort Wave and Extracorporeal Shock Wave: A Comparative Study. Med. Sci. Monit. Int. Med. J. Exp. Clin. Res..

[59] Peixoto K.O., da Silva Bezerra A., Melo R.A., de Resende C.M.B.M., de Almeida E.O., Barbosa G.A.S. (2021). Short-Term Effect of Scalp Acupuncture on Pain, Sleep Disorders, and Quality of Life in Patients with Temporomandibular Disorders: A A Randomized Clinical Trial. Pain Med..

[60] Rady N.A., Bahgat M.M., Abdel-Hamid A.M. (2022). Promising Minimally Invasive Treatment Modalities for Symptomatic Temporomandibular Joint Disc Displacement with Reduction: A Randomized Controlled Clinical Trial. BMC Oral Health.

[61] Liu S.-S., Xu L.-L., Liu L.-K., Lu S.-J., Cai B. (2023). Platelet-Rich Plasma Therapy for Temporomandibular Joint Osteoarthritis: A Randomized Controlled Trial. J. Cranio-Maxillofac. Surg..

[62] Barbosa M.A., Tahara A.K., Ferreira I.C., Intelangelo L., Barbosa A.C. (2019). Effects of 8 Weeks of Masticatory Muscles Focused Endurance Exercises on Women with Oro-Facial Pain and Temporomandibular Disorders: A Placebo Randomised Controlled Trial. J. Oral Rehabil..

[63] Abbasgholizadeh Z.S., Evren B., Ozkan Y. (2020). Evaluation of the Efficacy of Different Treatment Modalities for Painful Temporomandibular Disorders. Int. J. Oral Maxillofac. Surg..

[64] Young A., Gallia S., Ryan J.F., Kamimoto A., Korczeniewska O.A., Kalladka M., Khan J., Noma N. (2021). Diagnostic Tool Using the Diagnostic Criteria for Temporomandibular Disorders: A Randomized Crossover-Controlled, Double-Blinded, Two-Center Study. J. Oral Facial Pain Headache.

[65] De la Torre Canales G., Câmara-Souza M.B., Poluha R.L., de Figueredo O.M.C., de Souza Nobre B.B., Ernberg M., Conti P.C.R., Rizzatti-Barbosa C.M. (2022). Long-Term Effects of a Single Application of Botulinum Toxin Type A in Temporomandibular Myofascial Pain Patients: A Controlled Clinical Trial. Toxins.

[66] Huth K.C., Bex A., Kollmuss M., Wuersching S.N. (2023). Recording the Maxillomandibular Relationship with the Aqualizer System Prior to Occlusal Splint Therapy for Treating Temporomandibular Disorders: A Randomized Controlled Trial. Sci. Rep..

[67] da Fonseca Rodrigues M., Rodrigues M.L., Bueno K.S., Aroca J.P., Camilotti V., Busato M.C.A., Mendonça M.J. (2019). Effects of Low-Power Laser Auriculotherapy on the Physical and Emotional Aspects in Patients with Temporomandibular Disorders: A Blind, Randomized, Controlled Clinical Trial. Complement. Ther. Med..

[68] Özden M.C., Atalay B., Özden A.V., Çankaya A., Kolay E., Yıldırım S. (2020). Efficacy of Dry Needling in Patients with Myofascial Temporomandibular Disorders Related to the Masseter Muscle. CRANIO^®^.

[69] Bayramoğlu Z., Tozoğlu S. (2021). Comparison of Single- and Double-Puncture Arthrocentesis for the Treatment of Temporomandibular Joint Disorders: A Six-Month, Prospective Study. CRANIO^®^.

[70] Brandão R.D.A.F.S., Mendes C.M.C., Brandão Filho R.A., De Sena E.P. (2022). Isotonic Exercises and Relaxing Techniques in Individuals with Temporomandibular Dysfunction. CRANIO^®^.

[71] Sun J., Zhu H., Lu C., Zhao J., Nie X., Yang Z., He D. (2023). Temporomandibular Joint Disc Repositioning and Occlusal Splint for Adolescents with Skeletal Class II Malocclusion: A Single-Center, Randomized, Open-Label Trial. BMC Oral Health.

[72] Chen Y.-Y., Fan H.-C., Tung M.-C., Chang Y.-K. (2019). The Association between Parkinson’s Disease and Temporomandibular Disorder. PLoS ONE.

[73] Costa Y.M., Ferreira D.M.A.O., Conti P.C.R., Baad-Hansen L., Svensson P., Bonjardim L.R. (2020). Topical Anaesthesia Degree Is Reduced in Temporomandibular Disorders Patients: A Novel Approach to Assess Underlying Mechanisms of the Somatosensory Alterations. J. Oral Rehabil..

[74] Şahin D., Kaya Mutlu E., Şakar O., Ateş G., İnan Ş., Taşkıran H. (2021). The Effect of the Ischaemic Compression Technique on Pain and Functionality in Temporomandibular Disorders: A Randomised Clinical Trial. J. Oral Rehabil..

[75] Koçer G., Şentürk M.F. (2022). Does the Cannula Diameter Affect Outcomes of Temporomandibular Joint (TMJ) Arthrocentesis?. J. Oral Maxillofac. Surg. Off. J. Am. Assoc. Oral Maxillofac. Surg..

[76] Ângelo D.F., Sanz D., Maffia F., Cardoso H.J. (2023). Outcomes of IncobotulinumtoxinA Injection on Myalgia and Arthralgia in Patients Undergoing Temporomandibular Joint Arthroscopy: A Randomized Controlled Trial. Toxins.

[77] Lei J., Yap A.U.-J., Liu M.-Q., Fu K.-Y. (2019). Condylar Repair and Regeneration in Adolescents/Young Adults with Early-Stage Degenerative Temporomandibular Joint Disease: A Randomised Controlled Study. J. Oral Rehabil..

[78] Melo R.A., de Resende C.M.B.M., de Figueirêdo Rêgo C.R., de Sousa Leite Bispo A., Barbosa G.A.S., de Almeida E.O. (2020). Conservative Therapies to Treat Pain and Anxiety Associated with Temporomandibular Disorders: A Randomized Clinical Trial. Int. Dent. J..

[79] Jo J.H., Jang Y., Chung G., Chung J.W., Park J.W. (2021). Long-Term Efficacy and Patient Satisfaction of Pulsed Radiofrequency Therapy in Temporomandibular Disorders. Medicine.

[80] Ritto F.G., Cueto A.P., dos Santos Canellas J.V., Zuniga J.R., Tiwana P.S., Pimentel T., Medeiros P.J. (2022). Arthrocentesis versus Nonsurgical Methods in the Management of Temporomandibular Joint Closed Lock and Pain: A Double-Blind Randomized Controlled Trial. Oral Surg. Oral Med. Oral Pathol. Oral Radiol..

[81] Jung W., Lee K.-E., Suh B.-J., Seok H., Lee D.-W. (2023). Deep Learning for Osteoarthritis Classification in Temporomandibular Joint. Oral Dis..

[82] Shimada A., Castrillon E.E., Svensson P. (2019). Revisited Relationships between Probable Sleep Bruxism and Clinical Muscle Symptoms. J. Dent..

[83] Kopacz Ł., Ciosek Ż., Gronwald H., Skomro P., Ardan R., Lietz-Kijak D. (2020). Comparative Analysis of the Influence of Selected Physical Factors on the Level of Pain in the Course of Temporomandibular Joint Disorders. Pain Res. Manag..

[84] de Resende C.M.B.M., de Oliveira Medeiros F.G.L., de Figueiredo Rêgo C.R., Bispo A.D.S.L., Barbosa G.A.S., de Almeida E.O. (2021). Short-Term Effectiveness of Conservative Therapies in Pain, Quality of Life, and Sleep in Patients with Temporomandibular Disorders: A Randomized Clinical Trial. Cranio J. Craniomandib. Pract..

[85] Grossmann E., Ferreira L.A., Poluha R.L., Setogutti E., Iwaki L.C.V., Iwaki Filho L. (2022). Comparison of Two Needles Arthrocentesis versus Double Needle Cannula Arthrocentesis in the Treatment of Temporomandibular Disc Displacement. CRANIO^®^.

[86] Serrano-Hernanz G., Angulo-Carrere T., Ardizone-García I., Svensson P., Álvarez-Méndez A.M. (2023). Pressure Release Technique versus Placebo Applied to Cervical and Masticatory Muscles in Patients with Chronic Painful Myofascial Temporomandibular Disorder: A Randomised Clinical Trial. J. Oral Rehabil..

[87] Louw W.F., Reeves K.D., Lam S.K.H., Cheng A.-L., Rabago D. (2019). Treatment of Temporomandibular Dysfunction with Hypertonic Dextrose Injection (Prolotherapy): A Randomized Controlled Trial with Long-Term Partial Crossover. Mayo Clin. Proc..

[88] Şen S., Orhan G., Sertel S., Schmitter M., Schindler H.J., Lux C.J., Giannakopoulos N.N. (2020). Comparison of Acupuncture on Specific and Non-Specific Points for the Treatment of Painful Temporomandibular Disorders: A Randomised Controlled Trial. J. Oral Rehabil..

[89] Ram H.K., Shah D.N. (2021). Comparative Evaluation of Occlusal Splint Therapy and Muscle Energy Technique in the Management of Temporomandibular Disorders: A Randomized Controlled Clinical Trial. J. Indian Prosthodont. Soc..

[90] Rezazadeh F., Esnaashari N., Azad A., Emad S. (2022). The Effects of Botulinum Toxin A Injection on the Lateral Pterygoid Muscle in Patients with a Painful Temporomandibular Joint Click: A Randomized Clinical Trial Study. BMC Oral Health.

[91] Cömert Kiliç S., Kiliç N., Güngörmüş M. (2023). Botulinum Toxin Versus Dextrose Prolotherapy: Which Is More Effective for Temporomandibular Joint Subluxation? A Randomized Clinical Trial. J. Oral Maxillofac. Surg. Off. J. Am. Assoc. Oral Maxillofac. Surg..

[92] Exposto F.G., Bendixen K.H., Ernberg M., Bach F.W., Svensson P. (2019). Characterization and Predictive Mechanisms of Experimentally Induced Tension-Type Headache. Cephalalgia Int. J. Headache.

[93] Leite W.B., Oliveira M.L., Ferreira I.C., Anjos C.F., Barbosa M.A., Barbosa A.C. (2020). Effects of 4-Week Diacutaneous Fibrolysis on Myalgia, Mouth Opening, and Level of Functional Severity in Women with Temporomandibular Disorders: A Randomized Controlled Trial. J. Manip. Physiol. Ther..

[94] de Assis Fonseca Santos Brandão R., Mendes C.M.C., da Silva Lopes T., Filho R.A.B., de Sena E.P. (2021). Neurophysiological Aspects of Isotonic Exercises in Temporomandibular Joint Dysfunction Syndrome. CoDAS.

[95] Gupta A.K., Gupta R., Gill S. (2022). Effectiveness of Vitamin D along with Splint Therapy in the Vit D Deficient Patients with Temporomandibular Disorder-A Randomized, Double-Blind, Placebo-Controlled Clinical Trial. J. Indian Prosthodont. Soc..

[96] de Macedo C.F., Sonza A., Puel A.N., dos Santos A.R. (2023). Trigger Point Dry Needling Increases Masseter Muscle Oxygenation in Patients with Temporomandibular Disorder. J. Appl. Oral Sci..

[97] Cahlin B.J., Lindberg C., Dahlström L. (2019). Cerebral Palsy and Bruxism: Effects of Botulinum Toxin Injections—A Randomized Controlled Trial. Clin. Exp. Dent. Res..

[98] Calixtre L.B., Oliveira A.B., Alburquerque-Sendín F., Armijo-Olivo S. (2020). What Is the Minimal Important Difference of Pain Intensity, Mandibular Function, and Headache Impact in Patients with Temporomandibular Disorders? Clinical Significance Analysis of a Randomized Controlled Trial. Musculoskelet. Sci. Pract..

[99] Cömert Kılıç S. (2021). Does Glucosamine, Chondroitin Sulfate, and Methylsulfonylmethane Supplementation Improve the Outcome of Temporomandibular Joint Osteoarthritis Management with Arthrocentesis plus Intraarticular Hyaluronic Acid Injection. A Randomized Clinical Trial. J. Cranio-Maxillofac. Surg..

[100] Aroca J.P., de Faveri Cardoso P.M., Favarão J., Zanini M.M., Camilotti V., Busato M.C.A., Mendonça M.J., Alanis L.R.A. (2022). Auricular Acupuncture in TMD—A Sham-Controlled, Randomized, Clinical Trial. Complement. Ther. Clin. Pract..

[101] Furquim L.R., Mélo A.M., Barbosa A.F.S., Olivato O.P., Silva-Sousa Y.T.C., Leite-Panissi C.R.A., Magri L.V. (2023). Application of Photobiomodulation for Chronic Pain-Related TMD on Pain Points versus Pre-Established Points: Randomized Clinical Trial. J. Photochem. Photobiol. B.

[102] Bhargava D., Thomas S., Pawar P., Jain M., Pathak P. (2019). Ultrasound-Guided Arthrocentesis Using Single-Puncture, Double-Lumen, Single-Barrel Needle for Patients with Temporomandibular Joint Acute Closed Lock Internal Derangement. Oral Maxillofac. Surg..

[103] Takeuchi-Sato T., Ono Y., Funato M., Sato H., Suganuma T., Baba K. (2020). Efficacy of an Email-Based Recording and Reminding System for Limiting Daytime Non-Functional Tooth Contact in Patients with Temporomandibular Disorders: A Randomized Controlled Trial. J. Oral Rehabil..

[104] Kış H.C., Soydan Çabuk D. (2021). Evaluation of Styloid Chain Calcification Related to Temporomandibular Joint Disc Displacement: A Retrospective Cohort Study. Oral Radiol..

[105] Işık G., Kenç S., Özveri Koyuncu B., Günbay S., Günbay T. (2022). Injectable Platelet-Rich Fibrin as Treatment for Temporomandibular Joint Osteoarthritis: A Randomized Controlled Clinical Trial. J. Cranio-Maxillofac. Surg..

[106] Fang X., Xiong X., Lin J., Wu Y., Xiang J., Wang J. (2023). Machine-Learning–Based Detection of Degenerative Temporomandibular Joint Diseases Using Lateral Cephalograms. Am. J. Orthod. Dentofac. Orthop..

[107] Saha F.J., Pulla A., Ostermann T., Miller T., Dobos G., Cramer H. (2019). Effects of Occlusal Splint Therapy in Patients with Migraine or Tension-Type Headache and Comorbid Temporomandibular Disorder. Medicine.

[108] Madani A., Ahrari F., Fallahrastegar A., Daghestani N. (2020). A Randomized Clinical Trial Comparing the Efficacy of Low-Level Laser Therapy (LLLT) and Laser Acupuncture Therapy (LAT) in Patients with Temporomandibular Disorders. Lasers Med. Sci..

[109] Alves G.Â.D.S., da Rocha Gondim Y.R., de Lima J.A.S., da Silva M.A.P., Florêncio D.S.F., de Almeida L.N.A., de Silva H.J. (2021). Effects of photobiomodulation associated with orofacial myofactional therapy on temporomandibular joint dysfunction. CoDAS.

[110] Zwiri A.M., Ahmad W.M.A.W., Asif J.A., Phaik K.S., Husein A., Kassim N.K., Ab-Ghani Z. (2022). A Randomized Controlled Trial Evaluating the Levels of the Biomarkers Hs-CRP, IL-6, and IL-8 in Patients with Temporomandibular Disorder Treated with LLLT, Traditional Conservative Treatment, and a Combination of Both. Int. J. Environ. Res. Public Health.

[111] Seyhan M., Atalay E.S. (2023). Is Core Stability Training Effective in Temporomandibular Disorder? A Randomized Controlled Trial. Clin. Oral Investig..

[112] Monaco A., Pietropaoli D., Cooper B.C., Ortu E., IAPNOR (2019). A Device Improves Signs and Symptoms of TMD. Pain Res. Manag..

[113] Zarate M.A., Frusso R.D., Reeves K.D., Cheng A.-L., Rabago D. (2020). Dextrose Prolotherapy Versus Lidocaine Injection for Temporomandibular Dysfunction: A Pragmatic Randomized Controlled Trial. J. Altern. Complement. Med..

[114] Gikić M., Vrbanović E., Zlendić M., Alajbeg I.Z. (2021). Treatment Responses in Chronic Temporomandibular Patients Depending on the Treatment Modalities and Frequency of Parafunctional Behaviour. J. Oral Rehabil..

[115] Detoni R., Hartz C.S., Fusatto E.L., Bicalho E., Nacimento-Moraes K.S.G., Rizzatti-Barbosa C.M., Lopes F.O.T. (2022). Relationship between Osteopathic Manipulative Treatment of the Temporomandibular Joint, Molar Shim and the Orthostatic Position: A Randomized, Controlled and Double Blinded Study. J. Bodyw. Mov. Ther..

[116] Gębska M., Dalewski B., Pałka Ł., Kiczmer P., Kołodziej Ł. (2023). Effect of Physiotherapeutic Procedures on the Bioelectric Activity of the Masseter Muscle and the Range of Motion of the Temporomandibular Joints in the Female Population with Chronic Pain: A Randomized Controlled Trial. BMC Oral Health.

[117] Gokçe Kutuk S., Gökçe G., Arslan M., Özkan Y., Kütük M., Kursat Arikan O. (2019). Clinical and Radiological Comparison of Effects of Platelet-Rich Plasma, Hyaluronic Acid, and Corticosteroid Injections on Temporomandibular Joint Osteoarthritis. J. Craniofac. Surg..

[118] Ghodrati M., Mosallanezhad Z., Shati M., Noroozi M., Moghadam A.N., Rostami M., Nourbakhsh M. (2020). Adding Temporomandibular Joint Treatments to Routine Physiotherapy for Patients with Non-Specific Chronic Neck Pain: A Randomized Clinical Study. J. Bodyw. Mov. Ther..

[119] Wahlund K., Nilsson I.-M., Carlsson A.D., Larsson B., Wänman A. (2021). Internet-Based Treatment for Adolescents with Symptomatic Temporomandibular Joint Disc Displacement with Reduction. A Randomized Controlled Clinical Trial. Acta Odontol. Scand..

[120] Ekici Ö., Dündar Ü., Büyükbosna M. (2022). Comparison of the Efficiency of High-Intensity Laser Therapy and Transcutaneous Electrical Nerve Stimulation Therapy in Patients with Symptomatic Temporomandibular Joint Disc Displacement with Reduction. J. Oral Maxillofac. Surg. Off. J. Am. Assoc. Oral Maxillofac. Surg..

[121] Martins I.S., Radaic P., Marchi L., Barreto G., Pastore G.P. (2023). Assessment of Postoperative Pain in Patients Undergoing Temporomandibular Joint Arthroscopy with Infiltration of Dexamethasone Disodium Phosphate in Different Concentrations. A Randomized Controlled Trial. J. Cranio-Maxillofac. Surg..

[122] Isacsson G., Schumann M., Nohlert E., Mejersjö C., Tegelberg Å. (2019). Pain Relief Following a Single-dose Intra-articular Injection of Methylprednisolone in the Temporomandibular Joint Arthralgia—A Multicentre Randomised Controlled Trial. J. Oral Rehabil..

[123] Monteiro L., Ferreira R., Resende T., Pacheco J.J., Salazar F. (2020). Effectiveness of Photobiomodulation in Temporomandibular Disorder-Related Pain Using a 635 Nm Diode Laser: A Randomized, Blinded, and Placebo-Controlled Clinical Trial. Photobiomodulation Photomed. Laser Surg..

[124] Castaño-Joaqui O.G., Cano-Sánchez J., Campo-Trapero J., Muñoz-Guerra M.F. (2021). TMJ Arthroscopy with Hyaluronic Acid: A 12-Month Randomized Clinical Trial. Oral Dis..

[125] Ekici Ö., Dündar Ü., Büyükbosna M. (2022). Effectiveness of High-Intensity Laser Therapy in Patients with Myogenic Temporomandibular Joint Disorder: A Double-Blind, Placebo-Controlled Study. J. Stomatol. Oral Maxillofac. Surg..

[126] Simões C.A.S.C., da Silva M.A.M., Magesty R.A., Falci S.G.M., Douglas-de-Oliveira D.W., Gonçalves P.F., Flecha O.D. (2023). Counselling Treatment versus Counselling Associated with Jaw Exercises in Patients with Disc Displacement with Reduction—A Single-Blinded, Randomized, Controlled Clinical Trial. BMC Oral Health.

[127] Altaweel A.A., Elsayed S.A.-H., Baiomy A.A.B.A., Abdelsadek S.E., Hyder A.A. (2019). Extraoral versus Intraoral Botulinum Toxin Type A Injection for Management of Temporomandibular Joint Disc Displacement with Reduction. J. Craniofac. Surg..

[128] Al Sayegh S., Vasilatou I., Kumar A., Al Barwari C., Fredriksson L., Grigoriadis A., Christidis N. (2020). Experimental Pain and Fatigue Induced by Excessive Chewing. BMC Oral Health.

[129] Costa D.R., Pessoa D.R., Seefeldt V.B., Costa D.R., Maia D.T.L., dos Santos Maciel T., Mota B.B.M., Delpasso C.A., Ribeiro C.A.D., Nicolau R.A. (2021). Orofacial Evaluation of Individuals with Temporomandibular Disorder after LED Therapy Associated or Not of Occlusal Splint: A Randomized Double-Blind Controlled Clinical Study. Lasers Med. Sci..

[130] de Sousa D.F.M., dos Santos Malavazzi T.C., Deana A.M., Horliana A.C.R.T., Fernandes K.P.S., Bussadori S.K., Mesquita-Ferrari R.A. (2022). Simultaneous Red and Infrared Light-Emitting Diodes Reduced Pain in Individuals with Temporomandibular Disorder: A Randomized, Controlled, Double-Blind, Clinical Trial. Lasers Med. Sci..

[131] Gonzalez-Perez L.-M., Vera-Martin R., Montes-Latorre E., Torres-Carranza E., Infante-Cossio P. (2023). Botulinum Toxin and Percutaneous Needle Electrolysis for the Treatment of Chronic Masticatory Myalgia. Toxins.

[132] Yilmaz O., Korkmaz Y.T., Tuzuner T. (2019). Comparison of Treatment Efficacy between Hyaluronic Acid and Arthrocentesis plus Hyaluronic Acid in Internal Derangements of Temporomandibular Joint. J. Cranio-Maxillofac. Surg..

[133] Lam J., Svensson P., Alstergren P. (2020). Internet-Based Multimodal Pain Program with Telephone Support for Adults with Chronic Temporomandibular Disorder Pain: Randomized Controlled Pilot Trial. J. Med. Internet Res..

[134] Miotto E., Salvatore Freitas K.M., Mori A.A., Valarelli F.P., Gobbi de Oliveira R.C., Oliveira R.C. (2021). Effect of Botulinum Toxin on Quality of Life of Patients with Chronic Myofascial Pain. Pain Manag..

[135] Dasukil S., Arora G., Boyina K.K., Jena A.K., Jose A., Das S. (2022). Intra-Articular Injection of Hyaluronic Acid versus Platelet-Rich Plasma Following Single Puncture Arthrocentesis for the Management of Internal Derangement of TMJ: A Double-Blinded Randomised Controlled Trial. J. Cranio-Maxillofac. Surg..

[136] Olbort C., Pfanne F., Schwahn C., Bernhardt O. (2023). Training of the Lateral Pterygoid Muscle in the Treatment of Temporomandibular Joint Disc Displacement with Reduction: A Randomised Clinical Trial. J. Oral Rehabil..

[137] Saranya B., Ahmed J., Shenoy N., Ongole R., Sujir N., Natarajan S. (2019). Comparison of Transcutaneous Electric Nerve Stimulation (TENS) and Microcurrent Nerve Stimulation (MENS) in the Management of Masticatory Muscle Pain: A Comparative Study. Pain Res. Manag..

[138] Serritella E., Scialanca G., Di Giacomo P., Di Paolo C. (2020). Local Vibratory Stimulation for Temporomandibular Disorder Myofascial Pain Treatment: A Randomised, Double-Blind, Placebo-Controlled Preliminary Study. Pain Res. Manag..

[139] Rauch A., Jahn F., Roesner A., Hahnel S., Schierz O. (2021). Impact of the DC/TMD Instructional Video on the Practical Skills of Undergraduate Students-A Single-Blinded, Randomized Controlled Trial. Eur. J. Dent. Educ. Off. J. Assoc. Dent. Educ. Eur..

[140] Guandalini L.S., Santos V.B., Tesoro M.G., Maurício A.B., Drehmer de Almeida Cruz E., de Lima Lopes J., Lopes C.T., Bottura Leite de Barros A.L. (2022). Cross-Cultural Adaptation for Brazil and Validity of a Guide to Assist Nursing Students’ Clinical Reasoning. Int. J. Nurs. Knowl..

[141] Cigerim L., Kaplan V. (2023). Analgesic Efficacy of Naproxen-Codeine, Naproxen+dexamethasone, and Naproxen on Myofascial Pain: A Randomized Double-Blind Controlled Trial. CRANIO^®^.

[142] Öhrnell Malekzadeh B., Johansson Cahlin B., Widmark G. (2019). Conservative Therapy versus Arthrocentesis for the Treatment of Symptomatic Disk Displacement without Reduction: A Prospective Randomized Controlled Study. Oral Surg. Oral Med. Oral Pathol. Oral Radiol..

[143] Herpich C.M., Leal-Junior E.C.P., Politti F., de Paula Gomes C.A.F., dos Santos Glória I.P., de Souza Amaral M.D.F.R., Herpich G., de Azevedo L.M.A., de Oliveira Gonzalez T., Biasotto-Gonzalez D.A. (2020). Intraoral Photobiomodulation Diminishes Pain and Improves Functioning in Women with Temporomandibular Disorder: A Randomized, Sham-Controlled, Double-Blind Clinical Trial. Lasers Med. Sci..

[144] Priyadarshini S., Gnanam A., Sasikala B., Elavenil P., Raja Sethupathy Cheeman S., Mrunalini R., Krishna Kumar Raja V.B. (2021). Evaluation of Prolotherapy in Comparison with Occlusal Splints in Treating Internal Derangement of the Temporomandibular Joint—A Randomized Controlled Trial. J. Cranio-Maxillofac. Surg..

[145] Asadpour N., Shooshtari Z., Kazemian M., Gholami M., Vatanparast N., Samieirad S. (2022). Combined Platelet-Rich Plasma and Hyaluronic Acid Can Reduce Pain in Patients Undergoing Arthrocentesis for Temporomandibular Joint Osteoarthritis. J. Oral Maxillofac. Surg. Off. J. Am. Assoc. Oral Maxillofac. Surg..

[146] Mohanty S., Shankar H., Chaudhary Z., Sharma P., Verma A., Gupta A. (2023). Does the Exploration of the Temporomandibular Joint Using a Deep Subfascial Technique Result in a Superior Operative Outcome?. J. Oral Maxillofac. Surg. Off. J. Am. Assoc. Oral Maxillofac. Surg..

[147] Dalewski B., Kamińska A., Szydłowski M., Kozak M., Sobolewska E. (2019). Comparison of Early Effectiveness of Three Different Intervention Methods in Patients with Chronic Orofacial Pain: A Randomized, Controlled Clinical Trial. Pain Res. Manag..

[148] De Giorgi I., Castroflorio T., Cugliari G., Deregibus A. (2020). Does Occlusal Splint Affect Posture? A Randomized Controlled Trial. CRANIO^®^.

[149] Lukic N., Saxer T., Hou M.-Y., Zumbrunn Wojczyńska A., Gallo L.M., Colombo V. (2021). Short-Term Effects of NTI-Tss and Michigan Splint on Nocturnal Jaw Muscle Activity: A Pilot Study. Clin. Exp. Dent. Res..

[150] da Silva Dias W.C.F.G., Cavalcanti R.V.A., Magalhães Júnior H.V., de Araújo Pernambuco L., dos Santos Alves G.Â. (2022). Effects of Photobiomodulation Combined with Orofacial Myofunctional Therapy on the Quality of Life of Individuals with Temporomandibular Disorder. CoDAS.

[151] Bhargava D. (2023). Is Single Puncture Superior to Double Puncture Arthrocentesis in Patients with Wilkes III Internal Derangement?. J. Oral Maxillofac. Surg. Off. J. Am. Assoc. Oral Maxillofac. Surg..

[152] Dolwick M.F., Diaz D., Freburg-Hoffmeister D.L., Widmer C.G. (2020). A Randomized, Double-Blind, Placebo-Controlled Study of the Efficacy of Steroid Supplementation after Temporomandibular Joint Arthrocentesis. J. Oral Maxillofac. Surg. Off. J. Am. Assoc. Oral Maxillofac. Surg..

[153] Magri L.V., Bataglion C., Leite-Panissi C.R.A. (2021). Follow-up Results of a Randomized Clinical Trial for Low-Level Laser Therapy in Painful TMD of Muscular Origins. Cranio J. Craniomandib. Pract..

[154] Sitnikova V., Kämppi A., Teronen O., Kemppainen P. (2022). Effect of Botulinum Toxin Injection on EMG Activity and Bite Force in Masticatory Muscle Disorder: A Randomized Clinical Trial. Toxins.

[155] Araidy S., Sudri S., Mirochnik R., El-Naaj I.A. (2023). TMJ Arthroscopic Level 1 vs Arthrocentesis in the Management of Internal Derangement of the Temporomandibular Joint. Quintessence Int..

[156] Alajbeg I.Z., Vrbanović E., Lapić I., Alajbeg I., Vuletić L. (2020). Effect of Occlusal Splint on Oxidative Stress Markers and Psychological Aspects of Chronic Temporomandibular Pain: A Randomized Controlled Trial. Sci. Rep..

[157] Roychoudhury A., Yadav P., Bhutia O., Kaur K., Dekyi T., Pandey R.M. (2021). Growth Outcome and Jaw Functions Are Better after Gap Arthroplasty Plus Costochondral Graft Reconstruction Than Gap Arthroplasty Alone in Pediatric Temporomandibular Joint Ankylosis Patients: A Cluster Randomized Controlled Trial. J. Oral Maxillofac. Surg. Off. J. Am. Assoc. Oral Maxillofac. Surg..

[158] Ertas U., Ascl Y.E., Yalcin E., Urvasizoglu G. (2022). Evaluation of Intermaxillary Fixation (IMF) Screw Therapy with Craniomandibular Index Analysis for Chronic Recurrent Dislocation in the Temporomandibular Joint. Niger. J. Clin. Pract..

[159] Bayramoglu Z., Yavuz G.Y., Keskinruzgar A., Koparal M., Kaya G.S. (2023). Does Intra-Articular Injection of Tenoxicam after Arthrocentesis Heal Outcomes of Temporomandibular Joint Osteoarthritis? A Randomized Clinical Trial. BMC Oral Health.

[160] Nikolopoulou M., Aarab G., Ahlberg J., Hamburger H.L., de Lange J., Lobbezoo F. (2020). Oral Appliance Therapy versus Nasal Continuous Positive Airway Pressure in Obstructive Sleep Apnea: A Randomized, Placebo-Controlled Trial on Temporomandibular Side-Effects. Clin. Exp. Dent. Res..

[161] Shousha T., Alayat M., Moustafa I. (2021). Effects of Low-Level Laser Therapy versus Soft Occlusive Splints on Mouth Opening and Surface Electromyography in Females with Temporomandibular Dysfunction: A Randomized-Controlled Study. PLoS ONE.

[162] De la Torre Canales G., Poluha R.L., Pinzón N.A., Da Silva B.R., Almeida A.M., Ernberg M., Manso A.C., Bonjardim L.R., Rizzatti-Barbosa C.M. (2022). Efficacy of Botulinum Toxin Type-A I in the Improvement of Mandibular Motion and Muscle Sensibility in Myofascial Pain TMD Subjects: A Randomized Controlled Trial. Toxins.

[163] Tang Y.H., Vos L.M., Tuin A.J., Huddleston Slater J.J.R., Gareb B., van Bakelen N.B., Spijkervet F.K.L. (2023). Arthrocentesis versus Non-Surgical Intervention as Initial Treatment for Temporomandibular Joint Arthralgia: A Randomized Controlled Trial with Long-Term Follow-Up. Int. J. Oral Maxillofac. Surg..

[164] Tchivileva I.E., Hadgraft H., Lim P.F., Di Giosia M., Ribeiro-Dasilva M., Campbell J.H., Willis J., James R., Herman-Giddens M., Fillingim R.B. (2020). Efficacy and Safety of Propranolol for Treatment of Temporomandibular Disorder Pain: A Randomized, Placebo-Controlled Clinical Trial. Pain.

[165] Plaza-Manzano G., Delgado-de-la-Serna P., Díaz-Arribas M.J., Rodrigues-de-Souza D.P., Fernández-de-las-Peñas C., Alburquerque-Sendín F. (2021). Influence of Clinical, Physical, Psychological, and Psychophysical Variables on Treatment Outcomes in Somatic Tinnitus Associated with Temporomandibular Pain: Evidence from a Randomized Clinical Trial. Pain Pract..

[166] Haggag M.A., Al-Belasy F.A., Said Ahmed W.M. (2022). Dextrose Prolotherapy for Pain and Dysfunction of the TMJ Reducible Disc Displacement: A Randomized, Double-Blind Clinical Study. J. Cranio-Maxillofac. Surg..

[167] Hegab A.F., Hameed H.I.A.A., Hassaneen A.M., Hyder A. (2023). Synergistic Effect of Platelet Rich Plasma with Hyaluronic Acid Injection Following Arthrocentesis to Reduce Pain and Improve Function in TMJ Osteoarthritis. J. Stomatol. Oral Maxillofac. Surg..

[168] Montes-Carmona J.-F., Gonzalez-Perez L.-M., Infante-Cossio P. (2021). Treatment of Localized and Referred Masticatory Myofascial Pain with Botulinum Toxin Injection. Toxins.

[169] Araújo Oliveira Buosi J., Abreu Nogueira S.M., Sousa M.P., Soraya Costa Maia C., Regis R.R., de Freitas Pontes K.M., Bonjardim L.R., Sales Pinto Fiamengui L.M. (2021). Gluten-Free Diet Reduces Pain in Women with Myofascial Pain in Masticatory Muscles: A Preliminary Randomized Controlled Trial. J. Oral Facial Pain Headache.

[170] Ekici Ö., Dündar Ü., Gökay G.D., Büyükbosna M. (2022). Evaluation of the Efficiency of Different Treatment Modalities in Individuals with Painful Temporomandibular Joint Disc Displacement with Reduction: A Randomised Controlled Clinical Trial. Br. J. Oral Maxillofac. Surg..

[171] Potewiratnanond P., Vanichanon P., Limpuangthip N. (2023). Occlusal Splint and Combined Multiwave Locked System Laser Therapy Demonstrated Differential Patient-Reported Outcomes and Clinical Parameters: A Randomized Controlled Trial in Patients with Temporomandibular Disorder. J. Oral Rehabil..

[172] De la Torre Canales G., Alvarez-Pinzon N., Muñoz-Lora V.R.M., Vieira Peroni L., Farias Gomes A., Sánchez-Ayala A., Haiter-Neto F., Manfredini D., Rizzatti-Barbosa C.M. (2020). Efficacy and Safety of Botulinum Toxin Type A on Persistent Myofascial Pain: A Randomized Clinical Trial. Toxins.

[173] De la Torre Canales G., Poluha R.L., Alvarez Pinzón Y.N., Rodrigues Conti P.C., Manfredini D., Sánchez-Ayala A., Rizzatti-Barbosa C.M. (2021). Effects of Botulinum Toxin Type A on the Psychosocial Features of Myofascial Pain TMD Subjects: A Randomized Controlled Trial. J. Oral Facial Pain Headache.

[174] Hyder A., Tawfik B.E., Elmohandes W. (2022). Efficacy of Computer-Guided versus Conventional Sodium Hyaluronate Injection in Superior Joint Space in Treatment of Temporomandibular Joint (TMJ) Internal Derangement: Comparative Randomized Controlled Trial. J. Stomatol. Oral Maxillofac. Surg..

[175] Işık G., Kenç S., Özveri Koyuncu B., Günbay S., Günbay T. (2023). Does the Use of Injectable Platelet-Rich Fibrin after Arthrocentesis for Disc Displacement without Reduction Improve Clinical Outcomes?. J. Oral Maxillofac. Surg. Off. J. Am. Assoc. Oral Maxillofac. Surg..

[176] Nambi G., Abdelbasset W.K. (2020). Efficacy of Maitland Joint Mobilization Technique on Pain Intensity, Mouth Opening, Functional Limitation, Kinesiophobia, Sleep Quality and Quality of Life in Temporomandibular Joint Dysfunction Following Bilateral Cervicofacial Burns. Burns.

[177] Slade G.D., Fillingim R.B., Ohrbach R., Hadgraft H., Willis J., Arbes S.J., Tchivileva I.E. (2021). COMT Genotype and Efficacy of Propranolol for TMD Pain: A Randomized Trial. J. Dent. Res..

[178] Grossmann E., Poluha R.L. (2022). Double-Puncture Versus Single-Puncture Arthrocentesis: A Randomized Controlled Trial with 3 Years of Follow-Up. J. Oral Facial Pain Headache.

[179] El-Shaheed N.H., Mostafa A.Z.H., Aboelez M.A. (2023). Efficacy of Stabilisation Splint and Low-Level Laser Therapy for Patients with Chronic Closed Lock from Non-Reducible Displaced Temporo-Mandibular Joint Discs: A Parallel Randomised Clinical Trial. J. Oral Rehabil..

[180] de Salles-Neto F.T., de Paula J.S., de Azevedo José Romero J.G., Almeida-Leite C.M. (2020). Acupuncture for Pain, Mandibular Function and Oral Health-Related Quality of Life in Patients with Masticatory Myofascial Pain: A Randomised Controlled Trial. J. Oral Rehabil..

[181] Keerthana S.R., Mohammed H.S., Hariprasad A., Anand M., Ayesha S. (2021). Comparative Evaluation of Condylar Guidance Obtained by Three Different Interocclusal Recording Materials in a Semi-Adjustable Articulator and Digital Panoramic Radiographic Images in Dentate Patients: An in Vivo Study. J. Indian Prosthodont. Soc..

[182] Nambi G., Abdelbasset W.K., Soliman G.S., Alessi A.A., Alsalem I.N., Ali Z.A. (2022). Clinical and Functional Efficacy of Gallium-Arsenide Super Pulsed Laser Therapy on Temporo Mandibular Joint Pain with Orofacial Myalgia Following Healed Unilateral Cervicofacial Burn—A Randomized Trial. Burns.

[183] Rodrigues C.A., de Oliveira Melchior M., Magri L.V., Mazzetto M.O. (2020). Can the Severity of Orofacial Myofunctional Conditions Interfere with the Response of Analgesia Promoted by Active or Placebo Low-Level Laser Therapy?. CRANIO^®^.

[184] Karadayi U., Gursoytrak B. (2021). Randomised Controlled Trial of Arthrocentesis with or without PRF for Internal Derangement of the TMJ. J. Cranio-Maxillofac. Surg. Off. Publ. Eur. Assoc. Cranio-Maxillofac. Surg..

[185] Ilczuk-Rypuła D., Zalewska M., Pietraszewska D., Dybek A., Nitecka-Buchta A., Postek-Stefańska L. (2022). Prevalence and Possible Etiological Factors of Molar-Incisor Hypomineralization (MIH) in Population of Silesian Children in Poland: A Pilot Retrospective Cohort Study. Int. J. Environ. Res. Public Health.

[186] Reynolds B., Puentedura E.J., Kolber M.J., Cleland J.A. (2020). Effectiveness of Cervical Spine High-Velocity, Low-Amplitude Thrust Added to Behavioral Education, Soft Tissue Mobilization, and Exercise for People with Temporomandibular Disorder with Myalgia: A Randomized Clinical Trial. J. Orthop. Sports Phys. Ther..

[187] Grossmann E., Poluha R.L. (2021). Comparison between TMJ Arthrocentesis Techniques with Different Needle Positions: A Randomized Single-Blind Controlled Clinical Trial. J. Cranio-Maxillofac. Surg. Off. Publ. Eur. Assoc. Cranio-Maxillofac. Surg..

[188] Kaur K., Roychoudhury A., Bhutia O., Bhalla A.S., Yadav R., Pandey R.M. (2020). Evaluation of Success of Transport Disc Distraction Osteogenesis and Costochondral Graft for Ramus Condyle Unit Reconstruction in Pediatric Temporomandibular Joint Ankylosis. J. Oral Maxillofac. Surg. Off. J. Am. Assoc. Oral Maxillofac. Surg..

[189] Tchivileva I.E., Ohrbach R., Fillingim R.B., Lim P.F., Giosia M.D., Ribeiro-Dasilva M., Campbell J.H., Hadgraft H., Willis J., Arbes S.J. (2021). Effect of Comorbid Migraine on Propranolol Efficacy for Painful TMD in a Randomized Controlled Trial. Cephalalgia.

[190] Shandilya S., Mohanty S., Sharma P., Chaudhary Z., Kohli S., Kumar R.D. (2020). Effect of Preoperative Intramuscular Injection of Botulinum Toxin A on Pain and Mouth Opening after Surgical Intervention in Temporomandibular Joint Ankylosis Cases: A Controlled Clinical Trial. J. Oral Maxillofac. Surg. Off. J. Am. Assoc. Oral Maxillofac. Surg..

[191] Bruti G., Atencio M.R., D’Urso A., Giacomo P.D., Paolo C.D. (2021). Okada Purifying Therapy in Combination with Duloxetine vs. Duloxetine Alone in Patients with TMD and Fibromyalgia: A Randomized Clinical Study. J. Complement. Integr. Med..

[192] Zhang Y., Zhang J., Wang L., Wang K., Svensson P. (2020). Effect of Transcutaneous Electrical Nerve Stimulation on Jaw Movement-Evoked Pain in Patients with TMJ Disc Displacement without Reduction and Healthy Controls. Acta Odontol. Scand..

[193] Lin Y.-P., Su Y.-H., Chin S.-F., Chou Y.-C., Chia W.-T. (2020). Light-Emitting Diode Photobiomodulation Therapy for Non-Specific Low Back Pain in Working Nurses. Medicine.

[194] Andrade N.N., Aggarwal N., Mathai P., Nerurkar S., Desai H., Gupta V. (2020). Is Dermis Fat Arthroplasty Better than Plain Gap Arthroplasty? A Prospective Randomised Controlled Trial. Br. J. Oral Maxillofac. Surg..

[195] Noordzij M., Dekker F.W., Zoccali C., Jager K.J. (2011). Sample Size Calculations. Nephron Clin. Pract..

[196] Das S., Mitra K., Mandal M. (2016). Sample Size Calculation: Basic Principles. Indian J. Anaesth..

[197] Pourhoseingholi M.A., Vahedi M., Rahimzadeh M. (2013). Sample Size Calculation in Medical Studies. Gastroenterol. Hepatol. Bed Bench.

[198] Ryan T.P. (2013). Sample Size Determination and Power.

[199] Rosner B. (2016). Fundamentals of Biostatistics.

[200] Cochran W.G. (1977). Sampling Techniques.

[201] Jafari M., Ansari-Pour N. (2019). Why, When and How to Adjust Your P Values?. Cell J. Yakhteh.

[202] Miller J., Ulrich R. (2019). The Quest for an Optimal Alpha. PLoS ONE.

[203] Livingston E.H., Cassidy L. (2005). Statistical Power and Estimation of the Number of Required Subjects for a Study Based on the T-Test: A Surgeon’s Primer. J. Surg. Res..

[204] Dorey F.J. (2011). In Brief: Statistics in Brief: Statistical Power: What Is It and When Should It Be Used?. Clin. Orthop..

[205] Gent D.H., Esker P.D., Kriss A.B. (2018). Statistical Power in Plant Pathology Research. Phytopathology.

[206] Fox N., Mathers N. (1997). Empowering Research: Statistical Power in General Practice Research. Fam. Pract..

[207] Bababekov Y.J., Hung Y.-C., Hsu Y.-T., Udelsman B.V., Mueller J.L., Lin H.-Y., Stapleton S.M., Chang D.C. (2019). Is the Power Threshold of 0.8 Applicable to Surgical Science?-Empowering the Underpowered Study. J. Surg. Res..

[208] Johnston K.M., Lakzadeh P., Donato B.M.K., Szabo S.M. (2019). Methods of Sample Size Calculation in Descriptive Retrospective Burden of Illness Studies. BMC Med. Res. Methodol..

[209] Kim J., Seo B.S. (2013). How to Calculate Sample Size and Why. Clin. Orthop. Surg..

[210] Lakens D. (2013). Calculating and Reporting Effect Sizes to Facilitate Cumulative Science: A Practical Primer for t-Tests and ANOVAs. Front. Psychol..

[211] Tomczak M., Tomczak-Łukaszewska E. (2014). The Need to Report Effect Size Estimates Revisited. An Overview of Some Recommended Measures of Effect Size. TRENDS Sport Sci..

[212] Fritz C.O., Morris P.E., Richler J.J. (2012). Effect Size Estimates: Current Use, Calculations, and Interpretation. J. Exp. Psychol. Gen..

[213] Hedges L.V. (1981). Distribution Theory for Glass’s Estimator of Effect Size and Related Estimators. J. Educ. Stat..

[214] Delacre M., Lakens D., Ley C., Liu L., Leys C. (2023). Why Hedges’ G*s Based on the Non-Pooled Standard Deviation Should Be Reported with Welch’s t-Test.

[215] Stephanie Glass’s Delta. https://www.statisticshowto.com/glasss-delta/.

[216] Schmidt F.L. (1992). What Do Data Really Mean? Research Findings, Meta-Analysis, and Cumulative Knowledge in Psychology. Am. Psychol..

[217] Hedge’s g Statistic. https://www.itl.nist.gov/div898/software/dataplot/refman2/auxillar/hedgeg.htm.

[218] Effect Size Calculator—Cohen’s d, Cohen’s h, Phi(φ), R Squared, Eta Squared. https://www.statskingdom.com/effect-size-calculator.html.

[219] Zieliński G., Matysik-Woźniak A., Baszczowski M., Rapa M., Ginszt M., Pająk B., Szkutnik J., Rejdak R., Gawda P. (2023). Myopia & Painful Muscle Form of Temporomandibular Disorders: Connections between Vision, Masticatory and Cervical Muscles Activity and Sensitivity and Sleep Quality. Sci. Rep..

[220] Brydges C.R. (2019). Effect Size Guidelines, Sample Size Calculations, and Statistical Power in Gerontology. Innov. Aging.

[221] Gaeta L., Brydges C.R. (2020). An Examination of Effect Sizes and Statistical Power in Speech, Language, and Hearing Research. J. Speech Lang. Hear. Res. JSLHR.

[222] Lovakov A., Agadullina E.R. (2021). Empirically Derived Guidelines for Effect Size Interpretation in Social Psychology. Eur. J. Soc. Psychol..

[223] Gignac G.E., Szodorai E.T. (2016). Effect Size Guidelines for Individual Differences Researchers. Personal. Individ. Differ..

[224] Chen H., Cohen P., Chen S. (2010). How Big Is a Big Odds Ratio? Interpreting the Magnitudes of Odds Ratios in Epidemiological Studies. Commun. Stat.—Simul. Comput..

[225] Funder D.C., Ozer D.J. (2019). Evaluating Effect Size in Psychological Research: Sense and Nonsense. Adv. Methods Pract. Psychol. Sci..

[226] Kim H.-Y. (2016). Statistical Notes for Clinical Researchers: Sample Size Calculation 1. Comparison of Two Independent Sample Means. Restor. Dent. Endod..

[227] Cohen J. (1988). Statistical Power Analysis for the Behavioral Sciences.

[228] Hedges L.V., Olkin I. (1985). Statistical Methods for Meta-Analysis.

[229] IBM Documentation. https://www.ibm.com/docs/en/cognos-analytics/11.1.0?topic=terms-cramrs-v.

[230] Daines R. LibGuides: Statistics Resources: Partial Eta Squared. https://resources.nu.edu/statsresources/eta.

[231] FAQ/EffectSize—CBU Statistics Wiki. https://imaging.mrc-cbu.cam.ac.uk/statswiki/FAQ/effectSize.

[232] Andrade C. (2020). Mean Difference, Standardized Mean Difference (SMD), and Their Use in Meta-Analysis: As Simple as It Gets. J. Clin. Psychiatry.

[233] Zach Three Ways to Calculate Effect Size for a Chi-Square Test. *Statology* 2020. https://www.statology.org/effect-size-chi-square/.

[234] Automated Interpretation of Indices of Effect Size. https://cran.r-project.org/web/packages/effectsize/vignettes/interpret.html.

[235] Wu X., Zheng W., Mu D., Li N. (2019). UTCPredictor: An Uncertainty-aware Novel Teaching Cases Predictor. Comput. Appl. Eng. Educ..

[236] Richardson J. (2011). Eta Squared and Partial Eta Squared as Measures of Effect Size in Educational Research. Educ. Res. Rev..

[237] Sawilowsky S.S. (2009). New Effect Size Rules of Thumb. J. Mod. Appl. Stat. Methods.

[238] Serdar C.C., Cihan M., Yücel D., Serdar M.A. (2021). Sample Size, Power and Effect Size Revisited: Simplified and Practical Approaches in Pre-Clinical, Clinical and Laboratory Studies. Biochem. Medica.

[239] Sprent P. (2003). Statistics in Medical Research. Swiss Med. Wkly..

[240] van den Berg L., Lobatto R.M., Zuurmond W.W., de Lange J.J., Wagemans M.F., Bezemer P.D. (1997). Patients’ Refusal to Participate in Clinical Research. Eur. J. Anaesthesiol..

[241] Blumenthal-Barby J.S. (2017). “That’s the Doctor’s Job”: Overcoming Patient Reluctance to Be Involved in Medical Decision Making. Patient Educ. Couns..

[242] Kearney M., Downing M., Gignac E.A. (2024). Research Integrity and Academic Medicine: The Pressure to Publish and Research Misconduct. J. Osteopath. Med..

