# Correction: Zieliński, G.; Gawda, P. Analysis of the Use of Sample Size and Effect Size Calculations in a Temporomandibular Disorders Randomised Controlled Trial—Short Narrative Review. *J. Pers. Med.* 2024, *14*, 655

**DOI:** 10.3390/jpm15050188

**Published:** 2025-05-06

**Authors:** Grzegorz Zieliński, Piotr Gawda

**Affiliations:** Department of Sports Medicine, Medical University of Lublin, 20-093 Lublin, Poland

## Error in Table

In the original publication [1], an error occurred in the results presented in Table 2. In the study by Aguiar et al. [2], cited as reference 51, a score of 0 was assigned for sample size calculation. However, these calculations were present in the study. This results in a change in the penultimate column from 0 to 1. The corrected Table 2 appears below.

In the ‘Total In Year’ results, the value changes from 25 to 26, which increases the percentage from 81% to 84%. In ‘Total SS calculation’, the value changes from n = 94 to n = 95, without affecting the overall percentage in ‘Total SS’. This is because, for n = 94, the exact result was 57.6687, rounded to 58%, and now, for n = 95, the exact result is 58.2822, which also rounds to 58%.

## Text Correction and Error in Figure

Additionally, there is a change in the description ‘The sample analysed consisted of’ from 81 to 82 papers published in Q1. In Figure 1, one value changes from 69% to 70%. The corrected Figure 1 appears below.

The authors state that the scientific conclusions are unaffected. This correction was approved by the Academic Editor. The original publication has also been updated.

## Figures and Tables

**Figure 1:**
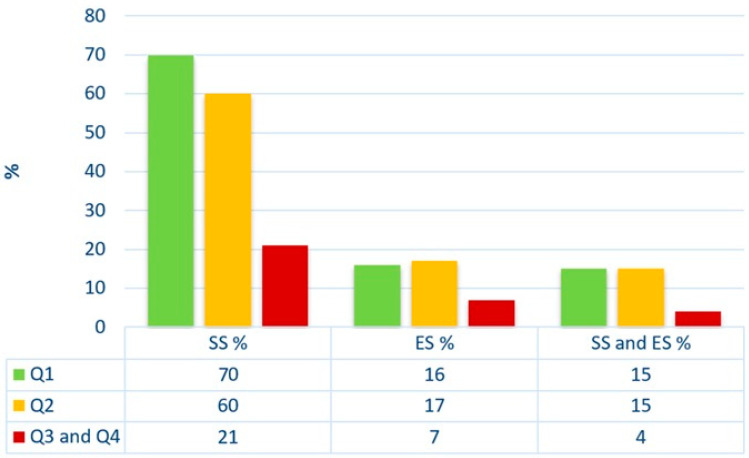
Analysis of the usage of SS, ES, and the combined use of SS and ES depending on the quartile determined by Scimago Journal & Country Rank. SS—sample size calculations; ES—effect size; Q—Quartiles according to Scimago Journal & Country Rank.

**Table 2:** Presentation of results on the use of sample size calculations and effect size in the analysed studies.

		2019				2020				2021				2022				2023		
No.	ID	Q	SS	ES	ID	Q	SS	ES	ID	Q	SS	ES	ID	Q	SS	ES	ID	Q	SS	ES
1	[32]	Q1	1	0	[33]	Q3	0	0	[34]	Q2	0	0	[35]	Q3	0	0	[36]	Q2	1	0
2	[37]	Q2	1	0	[38]	Q3	0	0	[39]	Q3	0	0	[40]	Q4	0	0	[41]	Q1	1	0
3	[42]	Q1	1	1	[43]	Q2	1	1	[44]	Q1	0	0	[45]	Q2	1	1	[46]	Q1	1	0
4	[47]	Q1	0	0	[48]	Q1	1	0	[49]	Q1	1	0	[50]	Q1	0	0	[51]	Q1	1	0
5	[52]	Q3	0	0	[53]	Q1	0	0	[54]	Q2	1	0	[55]	Q3	1	0	[56]	Q1	1	0
6	[57]	Q1	0	0	[58]	Q2	0	0	[59]	Q2	0	0	[60]	Q1	1	0	[61]	Q1	1	0
7	[62]	Q1	1	0	[63]	Q1	1	0	[64]	Q2	0	0	[65]	Q2	0	0	[66]	Q1	1	1
8	[67]	Q1	1	0	[68]	Q3	0	0	[69]	Q3	0	0	[70]	Q3	0	1	[71]	Q1	1	0
9	[72]	Q1	0	0	[73]	Q1	0	1	[74]	Q1	1	1	[75]	Q2	0	0	[76]	Q1	0	0
10	[77]	Q1	1	0	[78]	Q1	1	0	[79]	Q3	1	0	[80]	Q2	1	0	[81]	Q1	0	0
11	[82]	Q1	0	0	[83]	Q2	0	0	[84]	Q3	1	1	[85]	Q3	0	0	[86]	Q1	1	1
12	[87]	Q1	0	0	[88]	Q1	0	0	[89]	Q3	0	0	[90]	Q1	0	0	[91]	Q1	1	0
13	[92]	Q1	1	0	[93]	Q2	1	1	[94]	Q3	1	0	[95]	Q3	1	0	[96]	Q1	1	0
14	[97]	Q2	1	0	[98]	Q1	0	0	[99]	Q1	0	0	[100]	Q1	1	0	[101]	Q1	1	0
15	[102]	Q2	0	0	[103]	Q1	1	1	[104]	Q2	0	1	[105]	Q1	1	0	[106]	Q1	0	0
16	[107]	Q2	1	0	[108]	Q2	0	0	[109]	Q3	0	0	[110]	Q2	1	0	[111]	Q1	1	1
17	[112]	Q2	0	0	[113]	Q1	0	0	[114]	Q1	1	1	[115]	Q2	1	1	[116]	Q1	1	0
18	[117]	Q3	0	0	[118]	Q2	0	0	[119]	Q2	1	0	[120]	Q2	1	0	[121]	Q1	1	0
19	[122]	Q1	1	0	[123]	Q3	0	0	[124]	Q1	1	0	[125]	Q2	1	0	[126]	Q1	1	0
20	[127]	Q3	0	0	[128]	Q1	1	0	[129]	Q2	0	0	[130]	Q2	1	0	[131]	Q1	0	0
21	[132]	Q1	0	0	[133]	Q1	1	0	[134]	Q2	0	0	[135]	Q1	1	1	[136]	Q1	1	0
22	[137]	Q2	0	0	[138]	Q2	1	0	[139]	Q1	1	0	[140]	Q3	0	0	[141]	Q1	1	0
23	[142]	Q1	1	0	[143]	Q2	1	1	[144]	Q1	0	0	[145]	Q2	1	0	[146]	Q1	0	0
24	[147]	Q2	0	0	[148]	Q3	0	0	[149]	Q2	1	0	[150]	Q3	0	0	[151]	Q1	1	0
25					[152]	Q2	1	0	[153]	Q3	0	0	[154]	Q2	1	1	[155]	Q2	1	0
26					[156]	Q1	1	1	[157]	Q2	1	0	[158]	Q3	0	0	[159]	Q1	1	0
27					[160]	Q2	0	0	[161]	Q1	1	0	[162]	Q2	0	0	[163]	Q1	1	0
28					[164]	Q1	1	1	[165]	Q2	1	1	[166]	Q1	0	0	[167]	Q1	1	0
29					[168]	Q1	0	0	[169]	Q2	1	0	[170]	Q2	0	0	[171]	Q1	1	0
30					[172]	Q1	1	0	[173]	Q2	1	1	[174]	Q2	1	0	[175]	Q1	1	0
31					[176]	Q2	1	0	[177]	Q1	1	0	[178]	Q2	1	0	[179]	Q1	1	0
32					[180]	Q1	1	0	[181]	Q3	0	0	[182]	Q3	1	0				
33					[183]	Q3	0	0	[184]	Q1	1	0	[185]	Q2	0	0				
34					[186]	Q1	1	1	[187]	Q1	1	0								
35					[188]	Q2	1	0	[189]	Q1	1	1								
36					[190]	Q2	0	0	[191]	Q3	0	0								
37					[192]	Q1	0	0												
38					[193]	Q2	1	0												
39					[194]	Q2	1	0												
Total In Year			11	1			20	8			20	7			18	5			26	3
%			46	4			51	21			56	19			55	15			84	10
																				
		Total SS calculation									n	95			58	%				
		Total ES calculation									n	24			15	%				

SS—sample size calculations; ES—effect size; Q—Quartiles according to Scimago Journal & Country Rank.
